# The Genomic Characteristics of a Novel Partitivirus Infecting Industrial Hemp in Yunnan, China

**DOI:** 10.3390/microorganisms13122682

**Published:** 2025-11-25

**Authors:** Yuying Liu, Yanpin Xu, Xiaoxia Su, Fang Guan, Kuanyu Zheng, Xuan Chen

**Affiliations:** 1 Biotechnology and Germplasm Resources Research Institute, Yunnan Academy of Agricultural Sciences, Kunming 650205, China; liuyuying1220@outlook.com (Y.L.); sxx919@163.com (X.S.); gk797502@163.com (F.G.); 2 Yunnan Key Laboratory of Genetic Improvement of Herbal Oil Crops, Industrial Crops Research Institute, Yunnan Academy of Agricultural Sciences, Kunming 650205, China; cyn080328@126.com

**Keywords:** *Partitiviridae*, industrial hemp, genome, RdRp, CP

## Abstract

In this study, we identified a novel partitivirus infecting industrial hemp (*Cannabis sativa* L.), named as industrial hemp cryptic virus (IHCV). The complete genome sequence of IHCV comprises two RNA segments: dsRNA1 (1683 nt) encoding an RNA-dependent RNA polymerase (RdRp, 481 aa), and dsRNA2 (1669 nt) encoding a coat protein (CP, 417 aa). Comparative sequence analyses revealed that RdRp shares 68.10% amino acid similarity with the Citrullus lanatus cryptic virus (CiLCV), while the CP exhibits 35.80% similarity with the vitis cryptic virus (VCV). Moreover, phylogenetic analysis showed that both the RdRp and CP proteins of IHCV clustered together with the pepper cryptic virus 1 (PCV1), which belongs to the genus *Deltapartitivirus*. Seeds detection assays revealed seed infection rates ranging from 20% to 90% among different industrial hemp cultivars. This is the first report of a novel partitivirus virus infecting industrial hemp.

## 1. Introduction

Industrial hemp classified as *Cannabis sativa* L. has a low level of cannabinol (<0.3%), and is widely cultivated in Yunnan, Heilongjiang, Shanxi, and other regions in China. It is widely utilised in medicine, textiles and paper manufacturing, functional food additives, and cosmetics [1,2]. To date, more than 20 viruses have been found to infect *C. sativa* [3,4,5,6,7], including potato virus Y (PVY) from the genus *Potyvirus*, tobacco streak virus (TSV) from the genus *Ilarvirus*, cucumber mosaic virus (CMV) from the genus *Cucumovirus* [8,9,10], hop latent viroid (HLVd) from the genus *Pospiviroidae* [4], and beet curly top virus (BCTV) of *Geminiviridae* [3], as well as cannabis cryptic virus (CanCV) of the *Partitiviridae* [11]. Viral infection in *Cannabis* often results in symptoms such as yellowing, mottling, and mosaic patterns on the leaves, which can significantly reduce cannabis yield and quality [6].

The family *Partitiviridae* is divided into five genera: *Alphapartitivirus*, *Betapartitivirus*, *Cryspovirus*, *Deltapartitivirus*, and *Gammapartitivirus*. Most *Partitiviridae* family members typically possess two double-stranded RNA segments. DsRNA1 (1.5–2.5 Kbp) encodes the RNA-dependent RNA polymerase (RdRp), and dsRNA2 (1.2–2.4 Kbp) encodes the capsid protein (CP) [12,13,14]. Partitiviruses are isometric and consist of a non-enveloped viral particle that is 25–43 nm in diameter [14,15,16]. Partitiviruses have been shown to infect a wide range of hosts, including plants, fungi, and protozoa. Among them, the partitiviruses reported to be capable of infecting plants belong to three genera: *Alphapartitivirus*, *Betapartitivirus*, and *Deltapartitivirus* [17,18,19]. Plant-infecting partitiviruses are transmitted via pollen or seeds by intercellular mechanisms or vertically during cell division [17,19,20,21]. Previous research has demonstrated that all viruses of the genus *Deltapartitivirus*, such as the PCV1, can infect plants and are transmitted via pollen and ovules [22,23].

This study identified a novel partitivirus, provisionally named as industrial hemp cryptic virus (IHCV). The complete genome sequence of IHCV was determined by next-generation sequencing (NGS), RT-PCR, and RACE. Sequence and phylogenetic tree analyses were used to determine the classification status of IHCV. Seed detection assays revealed the presence of IHCV in seeds of five cannabis cultivars.

## 2. Materials and Methods

### 2.1. Plant Material

**Figure 1:**
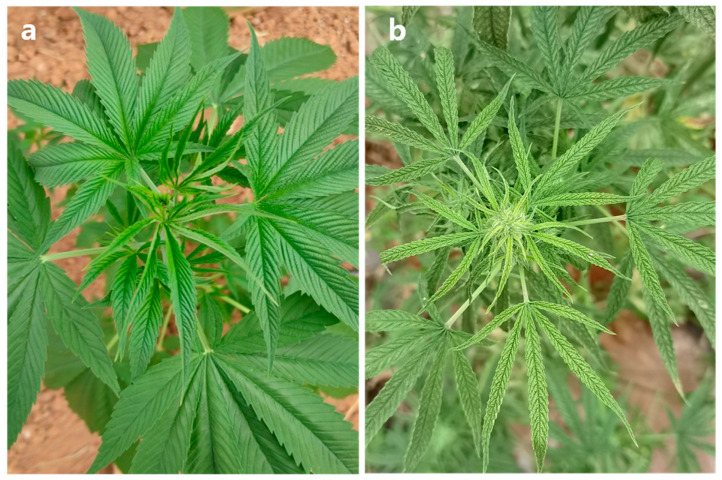
Virus-like symptoms on leaves of industrial hemp. (**a**) Asymptomatic leaves, (**b**) Symptomatic leaves exhibiting chlorosis and vein clearing.

In October 2023, industrial hemp plants exhibiting virus-like symptoms, such as chlorosis and vein clearing on leaves and plant dwarfing, were collected in Kunming, Yunnan Province, China (Figure 1). We collected four symptom samples, which were immediately frozen in liquid nitrogen and stored at −80 °C for subsequent high-throughput sequencing and RT-PCR experiment.

### 2.2. Next-Generation Sequencing

The total RNA of the samples was extracted using the mirVanami RNA Isolation Kit (Thermo Fisher Scientific, Madison, WI, USA), and rRNA was removed using the Ribo-Zero Plant Kit (Illumina, San Diego, CA, USA). The remaining RNA, after removing the ribosomal RNA, was reverse transcribed into cDNA using the HiFiScript gDNA Removal RT MasterMix (Cwbio, Beijing, China). Subsequently, next-generation sequencing (NGS) library construction and sequencing were carried out. NGS was carried out on the X Ten HiSeq Illumina sequencing platform (Illumina, San Diego, CA, USA). The raw reads were processed with Trimmomatic [24] to remove low-quality sequences and obtain clean reads. And the sequence was de novo assembled using the SPAdes software (SPAdes 3.15.4). The assembled sequences were annotated using the BlastN and BlastX tools from NCBI.

### 2.3. RT-PCR and RACE

Based on the contig sequence of the virus, several pairs of specific primers were designed by Primer 5 to amplify the viral target sequence and confirm the accuracy of the de novo assembled viral sequence (Table 1), and 50 μL PCR amplification reactions were conducted employing 25 μL 2× Hiffer^®^ Robust PCR Master Mix (Yeason, Shanghai, China), 1 μL reverse primer (10 pmol/L) and 1 μL forward primer (10 pmol/L), 5 μL of cDNA, and 18 μL ddH_2_O. The thermal cycling conditions were as follows: 3 min at 94 °C, followed by 33 cycles at 94 °C for 10 s, 56 °C for 20 s, 72 °C for 30 s; with a final extension step of 72 °C for 5 min, followed by 4 °C indefinitely. The amplified products were recovered via 1% agarose gel electrophoresis and subsequently sent to Beijing Tsingke Biotechnology Co., Ltd. (Kunming, China) for Sanger sequencing. DNAMAN 5.0 was used for the analysis, editing, and visualization of the sequencing results.

In addition, the 5′- and 3′-terminal sequences of the virus were amplified using the SMARTer RACE 5′/3′ Kit (Accurate Biology, Changsha, China), according to the designed 5′ and 3′ RACE-specific primers (Table 1), following the manufacturer’s instructions. For each 20 μL reaction, 3′/5′ RACE cDNA reaction mixtures consisting of 2 μL total RNA, 3′/5′ RACE RT primer, 8.5 μL/7.5 μL nuclease-free water, were combined, incubated at 72 °C for 3 min, then placed on ice for 2 min. The 11.5 μL 3′ RACE cDNA mixture and 10.5 μL 5′ RACE cDNA mixture were mixed with 4 μL 5× RACE RT buffer, 2 μL dNTP Mix, 0.5 μL RNase Inhibitor, and 2 μL Evo M-MLV RTase for RACE. In total, 1 µL of Template Switching Oligo was added only to the 5′ RACE cDNA reaction mixture. Final reaction mixtures (20 μL) were incubated at 42 °C for 90 min and 72 °C for 15 min. PCR amplification was performed using designed 5′ RACE and 3′ RACE-specific primers (Table 1). The purified DNA fragments were constructed on the pMD18-T vector and transformed into *Escherichia coli* DH5α cells for sequencing.

### 2.4. Sequence Analysis

Molecular phylogenetic analysis of partitiviruses was performed using MEGA 7.0. The Maximum Likelihood (ML) method with 1000 bootstrap replicates was applied to the RdRp and CP amino acid sequences. The RdRp phylogeny was constructed under the Le_Gascuel_2008 (LG) model with a discrete Gamma distribution (+G, 5 categories; parameter = 2.0853) and a proportion of invariant sites (+I, 2.51%). The CP phylogeny was based on the Whelan and Goldman model with Freq. (WAG+F) and a Gamma distribution (+G, 5 categories; parameter = 9.1168). Separately, recombination analysis of deltapartitiviruses was conducted in RDP4 using the RdRp and CP nucleotide sequences, employing the RDP, Geneconv, Chimaera, MaxChi, BootScan, SisScan, and 3Seq algorithms.

### 2.5. RT-PCR Detection in Seeds

Five industrial hemp cultivars (CY02, CY15, CY13, CY14, and CY08) were selected for the viral infection evaluation. Ten plants of each cultivar were randomly chosen for the test. Before the test, the surface of the seeds was disinfected (NaClO 0.1% 10 min). The total RNA of the seeds was extracted using TaKaRa MiniBEST Plant RNA Extraction Kit (TaKaRa, Dalian, China). RNA was reverse transcribed into cDNA, which referred to 2.2, and amplified from the seeds as described in Section 2.3, using the primers IHCV-RNA1-F/R.

## 3. Results

### 3.1. Viral Sequence Assembly

According to the NGS analysis results, 94.61 M raw reads were generated, yielding 93.67 M clean reads after removing low-quality reads (Q30 > 94.39%, N50 = 1051 bp). A total of 54,249 contigs were obtained by de novo assembly. BlastX and BlastN searches were conducted against NR databases, through which contig NODE_12332 (length 1578 nt) was mapped to the RNA1 of *Partitiviridae*, and NODE_12903 (length 1523 nt) was mapped to RNA2 of *Partitiviridae*. RT-PCR and RACE techniques were performed on symptomatic leaf samples, enabling the acquisition of the complete viral genome. Finally, the complete genomic sequence of this partitivirus was determined by RT-PCR amplification and RACE. The resultant dsRNA1 (NCBI accession No.PV554172) was 1683 bp in length, and dsRNA2 (NCBI accession No.PV554176) measured 1669 bp.

### 3.2. Viral Sequence Analysis

**Figure 2:**
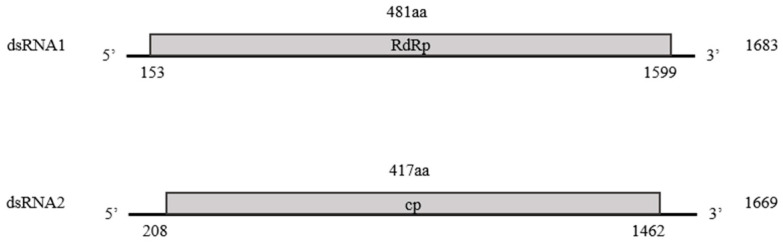
Schematic diagram of the genomic structure of the industrial hemp cryptic virus (IHCV). The shaded boxes indicate putative ORFs in dsRNA1 (RdRp) and dsRNA2 (CP) of IHCV.

Structurally, the genome of the identified partitivirus was similar to that of other *Partitiviridae* family members, with two dsRNA segments, dsRNA1 and dsRNA2. DsRNA1was 1683 nt, included a 153 bp 5′-UTR and an 84 bp 3′-UTR, and contained an ORF encoding a 481 aa RdRp with a molecular mass of 55.2 kDa. DsRNA2 was 1669 nt and comprised a 208 bp 5′-UTR and a 207 bp 3′-UTR, and harbored an ORF encoding a 417 aa CP with a molecular mass of 48.4 kDa (Figure 2). This genomic structure is consistent with that observed in the family *Partitiviridae.*

**Figure 3:**
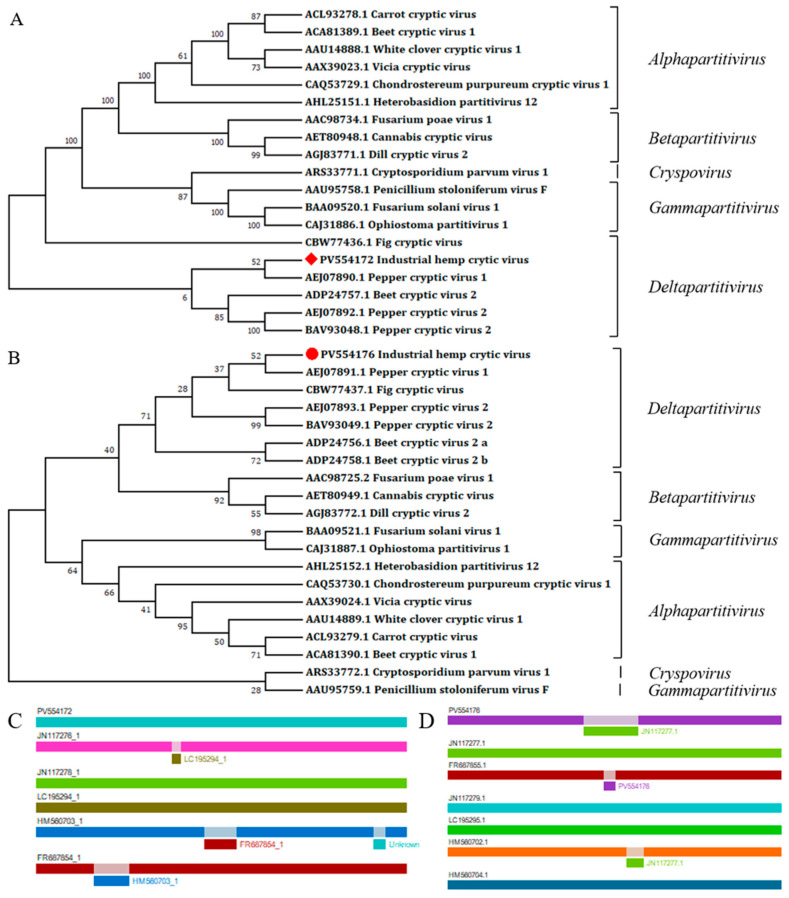
Molecular phylogenetic analyses of partitiviruses and recombination analyses of deltapartitiviruses. (**A**) Partitiviruses phylogenetic tree based on RdRp aa sequences; (**B**) partitiviruses phylogenetic tree based on CP aa sequences. The ML trees were conducted in MEGA 7.0 with 1000 bootstrap replicates, and the red square and the red dot are our sequences. (**C**) DsRNA1 recombinant clades suggested by the RDP4, (**D**) dsRNA2 recombinant clades suggested by the RDP4.

According to the nucleotide BlastN comparison results, dsRNA1 had the highest similarity with the CiLCV (PV701205.1), with 63.10% nt identity. DsRNA2 has the highest similarity with the Dactylorhiza cryptic virus 3 (DaCV3) (MT159308.1), sharing a 44.30% nt identity. The RdRp and CP aa sequences exhibited the highest similarity with CiLCV and VCV, respectively (68.10% and 35.80%) (Table 2). Through the ML method, phylogenetic analysis further grouped this virus alongside members of *Deltapartitivirus* (Figure 3A,B). Using RDP4 for interspecific recombination analysis of deltapartitiviruses revealed that recombination occurred in the dsRNA2 of IHCV within the 686–951 bp region. The minor parental sequence was identified as PCV1 (JN117277.1), while the major parental sequence was BCV-2 (HM560702.1). The value calculated by the RDP method was 3.34 × 10^−6^ (Figure 3C,D).

### 3.3. IHCV Detection in Seeds

**Figure 4:**
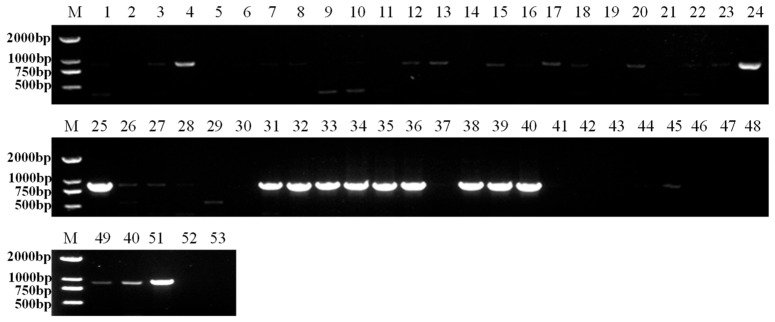
RT-PCR detection of IHCV on industrial hemp seeds of different cultivars. M: DL2000 marker, 1–10: CY02, 11–20: CY15, 21–30: CY13, 31–40: CY14, 41–50: CY08, 51: positive control, 52: negative control, 53: blank control.

Five industrial hemp cultivars (CY02, CY15, CY13, CY14, and CY08) were tested for IHCV infection in seeds, with detection rates ranging from 20% to 90% (Figure 4). Among them, cultivar CY14 exhibited the highest detection rate of 90%. Cultivar CY02 exhibited the lowest detection rate at 20%. The detection rates for cultivars CY15, CY13, and CY08 were 60%, 50%, and 40%, respectively.

## 4. Discussion

This study successfully obtained the full-length sequence of IHCV through NGS sequencing combined with RT-PCR and RACE techniques. Sequence analysis revealed that IHCV exhibited the highest similarity with CiLCV (68.1% of RdRp amino acid sequence) and VCV (35.80% of CP amino acid sequence), respectively. Phylogenetic analysis confirmed that this virus clusters with the genus *Deltapartitivirus* of the family *Partitiviridae*. According to the current ICTV classification criteria for a new species within the *Partitiviridae* (https://ictv.global/report/chapter/partitiviridae/partitiviridae), accessed on 1 September 2024, we propose that this virus infecting industrial hemp represents a novel species in this family.

In plants, partitiviruses persist indefinitely and coexist with host plants for extended periods. For instance, carnation cryptic virus (CarCV) was detected in *Dianthus*, persisting for 16 years, with neither thermotherapy nor meristem tip culture proving effective in eliminating the virus [20]. The detection rate of CanCV in the youngest fully expanded leaf of industrial hemp was 100% [11]. After 6–7 years of in vitro cultivation, beet cryptic virus -1/-2-/3 (BCV-1/BCV-2/BCV-3) was still detectable in the sugar beet (*Beta vulgaris* ssp. *Vulgaris*) [20].

Numerous plant-infecting partitiviruses have been reported to have seed-borne transmission capabilities via pollen and seeds [17,19]. For example, CanCV was identified as seed-transmissible in *Cannabis*, with a vertical transmission rate of 100% in offspring, regardless of the infected parent [11,25]. The CanCV infection rate of offspring from female non-infected sugar beet plants crossed with pollen from infected plants was 43%, while the reverse cross resulted in an 82% infection rate [19]. In this study, seed detection assays revealed that up to 90% of CY14 cultivar seeds were infected by IHCV. Seed propagation is an important aspect of industrial hemp, indicating the risk of IHCV transmission by seeds. Therefore, it is necessary to clarify the seed transmission characteristics and epidemiology of IHCV. We speculate that the reason for the variability in detection rates among these cultivars might be related to the reciprocal crossing of the parents.

To our knowledge, this is the first report of *Cannabis* infection by the novel partitivirus IHCV in China. So far, research on partitiviruses is very limited. Although partitiviruses lack a movement protein, they still interact with hosts, with adverse effects reported on specific on certain hosts. For instance, BCV resulted in a 20% decrease in sugar yield of sugar beets [26], while sclerotinia sclerotiorum partitivirus 1 (SsPV1) caused severe weakening of *A. thaliana* leaves [18], whereas some reports indicate that there are positive interactions between partitiviruses and hosts. For instance, the expression of white clover cryptic virus 1 (WCCV1) interfered with the formation of root nodules in Lotus japonicus [27]. Furthermore, given the seed-transmissible characteristics of patitiviruses, they can also be engineered into suitable delivery vectors for applications in crop gene function research, breeding, and cultivar improvement [28,29,30]. Consequently, further research is necessary to elucidate the transmission dynamics and pathogenic mechanisms of IHCV, as well as to develop effective management strategies.

## 5. Conclusions

Next-generation sequencing (NGS), RT-PCR, and RACE confirmed a novel partitivirus infecting industrial hemp, which was provisionally designated as industrial hemp cryptic virus (IHCV). Sequence analyses demonstrated that IHCV belongs to the family *Partitiviridae* and clustered with a member of the genus *Daltepartitivirus*. Seed detection assays confirmed the seed-carrying characteristics of IHCV in different cannabis cultivars. This is the first report of a novel partitivirus infecting industrial hemp. These findings have laid a foundation for further research on IHCV in cannabis breeding and cultivar improvement.

## Figures and Tables

**Table 1:** RT-PCR primers targeting the dsRNA sequences of IHCV.

Primer Name	Primer Sequence (5′-3′)	PCR Product Length (bp)
IHCV-RNA1-F	TGTTATAGACGTTGAGAACGGGT	1000
IHCV-RNA1-R	GATGTTCGTCCAAGGAAACTGAT	
IHCV-RNA2-1F	AACAGCAGACCCGCACAGGAATC	617
IHCV-RNA2-1R	TCGGCCAGTGTAGCTTGAGGAAA	
IHCV-RNA2-2F	TAACAGCAATGAAACACCTCAAAGT	896
IHCV-RNA2-2R	CTTCCTTAACGAAGATGAACTGTG	
3′RACE-IHCV-RNA1-F	TCCCGAATACCCTGTCGAAAC	310
3′RACE-IHCV-RNA2-F	ACAGACATTCACACCCAGTCAGCCT	200
5′RACE-IHCV-RNA1-R	ACCTCCTTTAGGTCCTTTCTCG	500
5′RACE-IHCV-RNA2-R	CATGTCGAGACGTAGATTCTAGCCG	330
Universal Short Primer	Manufacturer-provided	—

**Table 2:** Nucleotide (nt) and amino acid (aa) sequence similarity (%) of the RdRp and CP sequences of IHCV to other partitiviruses.

Virus	RdRp (IHCV)		CP (IHCV)	
	nt (%)	aa (%)	nt (%)	aa (%)
Citrulluslanatus cryptic virus	63.10	68.10	41.20	30.20
Dichroapartitivirus 2	62.60	64.50	43.10	35.60
Polygonatumpartitivirus 2	62.60	63.60	42.50	34.70
Alloteropsis cryptic virus 2	60.80	58.50	32.90	11.70
Dactylorhiza cryptic virus 3	59.80	55.90	44.30	33.50
Sinapis alba cryptic virus 1	59.60	59.50	42.30	29.10
Panax cryptic virus 1	59.60	59.70	40.50	30.40
Pepper cryptic virus 1	59.10	58.80	42.00	31.40
Vitis cryptic virus	56.60	64.40	25.90	35.80
Cannabis cryptic virus	46.00	18.70	45.80	18.10

## Data Availability

The original data presented in the study are openly available in [NCBI] at [accession number PV554172 and PV554176].
